# Association between COVID-19 infection and work exposure assessed by the Mat-O-Covid job exposure matrix in the CONSTANCES cohort

**DOI:** 10.1136/oemed-2022-108436

**Published:** 2022-09-20

**Authors:** Marc Fadel, Fabien Gilbert, Clément Legeay, Vincent Dubée, Yolande Esquirol, Catherine Verdun-Esquer, Aurelien Dinh, Grace Sembajwe, Marcel Goldberg, Yves Roquelaure, Annette Leclerc, Emmanuel Wiernik, Marie Zins, Alexis Descatha, Mat-O-Covid investigators, Alexis Descatha

**Affiliations:** 1 Univ Angers, CHU Angers, Univ Rennes, Inserm, EHESP, Irset (Institut de recherche en santé, environnement et travail) - UMR_S 1085, Ester Unit, SFR ICAT, CAPTV CDC, Angers, France; 2 Infection Control and Prevention Unit, CHU Angers, Angers, France; 3 Infectious and Tropical Diseases Department, University Hospital CHU Angers, Angers, France; 4 Immunology and New Concepts in ImmunoTherapy, INCIT, UMR 1302/EMR6001, Univ Angers, Nantes Université, INSERM, CNRS, Nantes, France; 5 Occupational and Environmental Health Department, CHU, CERPOP UMR 1295, Université Paul Sabatier Toulouse 3, Inserm, Toulouse, France; 6 Service Santé Travail Environnement, INSERM U1219, EPICENE, CHU de Bordeaux, Univ Bordeaux, Bordeaux, France; 7 Infectious Disease Unit, Raymond-Poincaré University Hospital, AP-HP (Paris Hospital), Paris Saclay University, Paris, France; 8 Department of Occupational Medicine, Epidemiology and Prevention, Donald and Barbara Zucker School of Medicine, Hofstra/ Northwell, Great Neck, New York, USA; 9 Unité "Cohortes en Population" UMS 011, Inserm/Université de Paris/Université Paris Saclay/UVSQ, Villejuif, France

**Keywords:** Epidemiology, COVID-19, Occupational Health, Public health

## Abstract

**Objectives:**

The COVID-19 pandemic has brought to light a new occupational health threat. We aimed to evaluate the association between COVID-19 infection and work exposure to SARS-CoV-2 assessed by a job-exposure matrix (JEM), in a large population cohort. We also estimated the population-attributable fraction among exposed subjects.

**Methods:**

We used the SAPRIS-SERO sample of the CONSTANCES cohort, limited to subjects actively working, and with a job code available and a questionnaire on extra work activities. The following outcomes were assessed: COVID-19 diagnosis was made by a physician; a seropositivity to the ELISA-S test (‘serology strict’) and ELISA-S test intermediate with positive ELISA-NP or a positive neutralising antibodies SN (‘serology large’). Job exposure was assessed using Mat-O-Covid, an expert-based JEM with an Index used as a continuous variable and a threshold at 13/1000.

**Results:**

The sample included 18 999 subjects with 389 different jobs, 47.7% were men with a mean age of 46.2 years (±9.2 years). The Mat-O-Covid index taken as a continuous variable or with a threshold greater than 13/1000 was associated with all the outcomes in bivariable and multivariable logistic models. ORs were between 1.30 and 1.58, and proportion of COVID-19 attributable to work among exposed participants was between 20% and 40%.

**Discussion:**

Using the Mat-O-Covid JEM applied to a large population, we found a significant association between work exposure to SARS-CoV-2 and COVID-19 infection, though the estimation of attributable fraction among exposed people remained low to moderate. Further studies during other exposed periods and with other methods are necessary.

**WHAT IS ALREADY KNOWN ON THIS TOPIC**

- Many jobs are at high risk of COVID-19 infection, such as healthcare workers.
- COVID-19 job-exposure matrices have been developed to consider SARS-CoV-2 exposure at work.

**WHAT THIS STUDY ADDS**

- Using the COVID-19 job-exposure Matrix ‘Mat-O-Covid’ applied to a large population, we found a significant association between work exposure to SARS-CoV-2 and COVID-19 infection.
- The estimation of attributable fraction among participants exposed at work exposure was low to moderate to SARS-CoV-2.

**HOW THIS STUDY MIGHT AFFECT RESEARCH, PRACTICE OR POLICY**

- Prevention measures implemented in the workplace might explain these results and emphasise the importance of occupational health and exposure research for other COVID-19 variants and workplace risks.

## Introduction

Since its first detection in the latter part of 2019 until this year, the novel COVID-19 caused by the SARS-CoV-2 has spread worldwide and infected millions of people, quickly becoming a tremendous global challenge for healthcare workers and policymakers. Front-line workers and now more broadly all workers are at risk of getting infected by SARS-CoV-2 at work, and occupational and public health units must deal with this new threat.^1–3^

Healthcare workers are clearly exposed to SARS-CoV-2 through their interaction with patients, but it is more difficult to assess occupational exposure to SARS-CoV-2 in non-healthcare settings. Many non-healthcare jobs are public-facing, resulting in varying levels of contact and close physical proximity to others, creating potential high-risk situations for infection.^4 5^ There are unresolved questions on exposure to SARS-CoV-2 attributable to work. A recent mortality study found that work confounding factors and mediating factors explained 20%–30% of the excess age-adjusted risks.^6^ However, such evaluations should include, not only mortality, but also infection rates (prevalence and incidence) and need a robust evaluation of the role of work exposure.

Job-exposure matrices (JEMs) were developed to address the intricacies of work exposure. The JEM is a tool in occupational medicine used to estimate exposure to various workplace risk factors and has been shown to produce reliable and cost-effective exposure estimates that minimise bias due to individual variability from self-reports. Estimating occupational exposure to SARS-CoV-2 has become increasingly important, especially with the spread of viral variants worldwide and the advent of long-term COVID-19 symptoms (often referred to as postacute sequelae of COVID-19 or postacute COVID-19 syndrome) and the need to apply adapted preventive strategies. On this basis, Mat-O-Covid was created as a JEM to evaluate workplace exposures to SARS-CoV-2.^7^

In these analyses, we aimed to apply the COVID-19 JEM Mat-O-Covid to the large population-based cohort CONSTANCES that had available information on COVID-19 infection, to assess the association between work exposure and COVID-19 infection. Using these results, we also aimed to estimate the population-attributable fraction among subjects exposed (AFE) to SARS-CoV-2 at work.

## Methods

### Population

CONSTANCES is a French general population-based cohort.^8^ More than 200 000 participants, aged 18–69 years, were recruited between 2012 and 2020 in 26 health screening centres across France. The recruitment was limited to people affiliated with the French National Health Insurance Fund that comprises active or former salaried workers and their families and excludes agricultural and self-employed workers.^8^ At enrolment, self-administered questionnaires were sent to participants to collect data on lifestyle, life events, health and occupation. Variables of interest were collected from the baseline self-administered questionnaire and medical interviews. For this work, we used a subsample of CONSTANCES participants who were included in the SAPRIS-SERO COVID-19 study,^9 10^ limited to subjects who answered to be at work, and with an available job code.

In this sample, each participant filled out an online questionnaire between June and October 2020. In addition to age (categorised as <30 years, 30–40 years, 40–50 years, 50–60 years and ≥60 years) and sex, participants answered questions on a potential COVID-19 infection diagnosed by a physician, and on the following activities since the end of the lockdown, coded in three categories (none; yes one time; yes more than one time): family gatherings, leisure activities, regular shopping, visiting public places. Data on usage of protective measures outside of work (hand sanitiser and mask-wearing) were also collected in three categories: no; yes, after almost every outing; yes, systematically after every outing (including not or never go out).

Mat-O-Covid is an expert-based JEM which uses the profession and socioprofessional categories ‘Catégories Socioprofessionnelles PCS 2003’ (French Classification of Occupations) as job codes in the dataset. To create the exposure index, a group of four experts in different occupational fields independently coded the data on occupational exposure to SARS-CoV-2 using 0 (low/no exposure) to 1 (likely/very frequent exposure)^11^ and other experts similarly coded prevention methods (see box 1). This resulted in an index for each job associated with the probability of exposure to SARS-CoV-2 at work, called the ‘Mat-O-Covid index’.^12^ The index was used as a continuous variable with a threshold defined in a previous study (ie, 13/1000, above which exposure has definitively occurred).^13^

Box 1 **Mat-O-Covid**

‘Mat-O-Covid’ is a job exposure matrix that was developed by a group of French experts to provide a global assessment of the probability of exposure and prevention of SARS-CoV2 only in the occupational field. This tool includes four elements that have been evaluated by experts, namely

**P1:**0%–100%, with as an example the probability of close direct contact: 5 as unlikely/very infrequent, 30 possible/ infrequent contact, 70 likely/very frequent contact.

**P2:**0%–100%, with examples of probability of effective prevention: 5 as unlikely/very infrequent, 30 possible/infrequent prevention, 70 probable/very frequent prevention.

**P3:**0%–100%, with an example of the probability of contact with an infected patient/virus: 5 as unlikely/very infrequent contact, 30 as possible/infrequent contact, 70 as likely/very frequent contact.

**P4:**0%–100%, with examples of probability of effective prevention: 5 as unlikely/very infrequent, 30 possible/infrequent prevention, 70 probable/very frequent prevention.

In a second step, it was necessary to calculate an overall probability of occupational contamination using four additional parameters:

- **F1**: Overall probability factor of exposure to SARS-CoV2 among non-diseased working people (public/colleagues). This factor was estimated to be 100.
- **F2**: Overall probability factor of exposure to SARS-CoV2 among sick patients encountered in the medicosocial sector. This factor was estimated at 10.
- **F3**: Global factor of probability of exposure to SARS-CoV2 of the profession and social category, all sectors of activity combined, in comparison with the medicosocial sector. This factor is between 1 and 10.
- **F4**: Global factor of virus circulation during the period considered. This factor is between 0 and 100.

For now, the F4 factor was evaluated at one for the moment.

From the above, an average probability of occupational SARS-CoV2 contamination assessed by the P5 formula, adding the probabilities of subject and patient contact, weighted by preventive measures and additional factors.

P5=[(P1x(1-P2)/F1)+(P3/F3x(1-P4)/F2]xF4x1000

The updated matrices are freely available in appendices of the papers (https://europepmc.org/article/pmc/pmc9091162 appendix 1 in French https://europepmc.org/articles/PMC9091162/bin/mmc1.xlsx, appendix 2 in English https://europepmc.org/articles/PMC9091162/bin/mmc2.xlsx).

The study participants also received an invitation to perform a serology test by dried-blood spot (DBS) self-sampling.^9^ Participants living in mainland France, who completed the questionnaires and who agreed to the serology test, received a DBS kit to be returned to the centralised biobank after capillary blood collection (CEPH Biobank, Paris, France). Two waves of kits were sent out: the first was a random sample of participants in 3 of the 12 mainland French regions, the second was extended to include all regions of France and all consenting participants. The Elisa test (Euroimmun, Lübeck, Germany) was used to detect anti-SARS-CoV-2 antibodies (IgG) directed against the S1 domain of the spike protein of the virus (ELISA-S). In accordance with the manufacturer’s instructions, a test was considered to be ELISA-S-positive if the optical density ratio was ≥1.1, ELISA-S indeterminate between 0.8 and 1.1, and ELISA-S-negative if <0.8. All samples with an ELISA-S test ≥0.7 were also tested with an ELISA test to detect IgG antibodies against the SARS-CoV-2 nucleocapsid protein (ELISA-NP) (Euroimmun, Lübeck, Germany), and with an in-house micro-neutralisation assay to detect neutralising anti-SARS-CoV-2 antibodies (SN), with a positive SN defined as a titre ≥40.^14^

Three outcomes were considered: (1) a reported infection diagnosed by a physician called ‘COVID-19 reported’, (2) seropositivity with the ELISA-S test only (ELISA-positive with an optical density ratio of ≥1.1.), called ‘Serology strict’, (3) ‘Serology strict’ or an indeterminate ELISA-S (between 0.8 and 1.1) with either a positive ELISA-NP or a positive SN, called ‘Serology large’.

Associations between the outcomes and the Mat-O-Covid index were calculated using univariable and multivariable logistic models adjusted for age, sex and extra work activities and use of protective measures outside of work. For extra work activities, the reference used was ‘yes, more than one time’ and for use of protective measures outside of work, ‘yes, systematically after every outing’. Differences of timing between questionnaires and serology were also considered in a two-category variable (3 months or more compared with less). All analyses were performed using Statistical Analysis System V.9.4 (SAS Institute). Using the index variable in two categories from the bivariable and multivariable analyses, we calculated the AFE individuals which estimated the proportion of cases of COVID-19 attributable to work in the industries and occupations at high risk^15^ : AFE = (OR – 1)/OR. Range was calculated using the 95% upper and lower limits of OR.

## Results

The sample with participants actively working included 18 999 subjects with 389 different jobs, with 47.7% of men and a mean age of 46.2 years (±9.2 years). Among them, 125 did not answer the COVID-19 question, 4437 lacked an Elisa test, and 4484 lacked an Elisa-SN and RN. Mean time between the questionnaire with occupation and the online questionnaire with COVID-19 information was of 2.8 months (±0.6) and mean time between the online questionnaire and serology was of 3.5 months (±1.0).

Before fall 2020, 613 subjects (3.3%) reported having COVID-19 diagnosed by a physician (‘COVID-19 reported’). In addition to being a woman and age between 30 and 40 years, Mat-O-Covid index was associated with ‘COVID-19 reported’, whereas subjects who reported no public places visits and use of hand sanitiser after almost every outing had a lower risk (table 1). Age (30–40 years), use of hand sanitiser after almost every outing, delayed questionnaire and Mat-O-Covid index remained significantly associated with ‘COVID-19 reported’ in multivariable analyses.

**Table 1:** Description of cases defined by reported COVID-19 by a physician and association with bivariable and multivariable analyses based on Mat-O-Covid index (continuous and categorised)

	Total	No of cases	Proportion (%)	Crude ORs (95% CI)	Adjusted ORs*, Mat-O-Covid index continuous (95% CI)	Adjusted ORs*, Mat-O-Covid index categorised (95% CI
Sex						
Men	9000	262	2.91	**1**	**1**	**1**
Women	9874	351	3.55	**1.23 (1.04 to 1.45)**	1.16 (0.95 to 1.43)	1.17 (0.95 to 1.44)
Age			3.11			
<30 years	483	15	3.91	**0.98 (0.58 to 1.68)**	**1.15 (0.61 to 2.16)**	**1.13 (0.60 to 2.12)**
30–40 years	4654	182	3.15	**1.25 (1.02 to 1.53)**	**1.32 (1.04 to 1.68)**	**1.32 (1.03 to 1.68)**
40–50 years	6818	215	3.05	**1**	**1**	**1**
50–60 years	5545	169	2.33	**0.97 (0.79 to 1.18)**	**1.03 (0.80 to 1.31)**	**1.03 (0.81 to 1.31)**
≥ 60 years	1374	32		**0.73 (0.50 to 1.07)**	**0.64 (0.38 to 1.06)**	**0.64 (0.39 to 1.07)**
Family meeting						
No	662	14	2.11	0.64 (0.38 to 1.10)	0.70 (0.36 to 1.37)	0.70 (0.36 to 1.37)
Yes, one time	1253	47	3.75	1.16 (0.85 to 1.57)	1.43 (1.00 to 2.06)	1.44 (1.00 to 2.06)
Yes, more than one time	16 880	550	3.26	1	1	1
Leisure activities						
No	4440	130	2.93	0.88 (0.72 to 1.07)	0.88 (0.68 to 1.14)	0.88 (0.68 to 1.13)
Yes, one time	1079	39	3.61	1.09 (0.78 to 1.52)	1.32 (0.90 to 1.94)	1.33 (0.91 to 1.95)
Yes, more than one time	13 272	441	3.32	1	1	1
Regular shopping						
No	369	6	1.63	0.49 (0.22 to 1.10)	0.51 (0.19 to 1.37)	0.51 (0.19 to 1.37)
Yes, one time	249	5	2.01	0.60 (0.25 to 1.47)	0.71 (0.26 to 1.93)	0.71 (0.26 to 1.94)
Yes, more than one time	18 165	596	3.28	1	1	1
Public places visit						
No	2889	77	2.67	**0.77 (0.60 to 0.98)**	0.75 (0.54 to 1.03)	0.75 (0.54 to 1.03)
Yes, one time	2468	69	2.8	**0.80 (0.62 to 1.04)**	0.74 (0.54 to 1.02)	0.74 (0.54 to 1.02)
Yes, more than one time	13 446	464	3.45	**1**	1	1
Use of hand sanitiser†						
No	389	19	4.88	**1.41 (0.88 to 2.26)**	**1.04 (0.52 to 2.06)**	**1.04 (0.52 to 2.05)**
Yes, after almost every outing	6208	164	2.64	**0.75 (0.62 to0.90)**	**0.74 (0.59 to 0.93)**	**0.74 (0.59 to 0.92)**
Yes, systematically after each outing (or I never go out)	12 212	428	3.5	**1**	**1**	**1**
Wearing mask†						
No	509	20	3.93	1.19 (0.74 to 1.91)	1.33 (0.75 to 2.34)	1.33 (0.75 to 2.34)
Yes, after almost every outing	13 599	434	3.19	0.96 (0.80 to 1.16)	0.96 (0.76 to 1.21)	0.96 (0.76 to 1.21)
Yes, systematically after each outing (or I never go out)	4696	156	3.32	1	1	1
Difference between questionnaires						
Less than 3 month	13 154	420	3.19	1	1	1
3 months or more	5720	193	3.37	1.06 (0.89 to 1.26)	1.04 (0.84 to 1.29)	1.04 (0.84 to 1.29)
Mat-O-Covid Index						
Continuous variable	18 874	613	3.25	**1.03 (1.01 to 1.04)**	**1.02 (1.01 to 1.03)**	
<13 per thousand	11 529	354	3.07	**1**		**1**
≥13 per thousand	1549	82	5.29	**1.76 (1.38 to 2.26)**		**1.58 (1.22 to 2.04)**

Bold results: overall test, p<0.05.

*Adjusted for sex, age, family meeting, leisure activities, regular shopping, public places visit, use of hand sanitiser, wearing mask and time between questionnaires.

†Outside of work

In the sample with ELISA serologies and ELISA-NP and SN testing (n=14 515), 1017 had a positive test (‘serology large’, 7.0%). In addition to being female and aged between 30 and 40 years, Mat-O-Covid index was associated with the ‘serology large’ outcome, whereas subjects older than 50 years and younger than 30 years, who reported no public places visited, use of hand sanitiser after almost every outing, and delayed questionnaire had a lower risk (table 2). Sex, age (less than 30 years or 50 years or more), use of hand sanitiser after almost every outing, delayed questionnaire and Mat-O-Covid index remained significantly associated with the ‘serology large’ outcome in multivariable analyses. In the sample with available ELISA testing (n=14 562), 911 had a positive test (‘serology strict’, 6.3%). A similar pattern of associations is observed in table 3.

**Table 2:** Description of cases defined by ‘serology large’ and association with bivariable and multivariable analyses based on Mat-O-Covid index (continuous and categorised)

	Total	No of cases	Proportion (%)	Crude ORs (95% CI)	Adjusted ORs*, Mat-O-Covid index continuous (95% CI)	Adjusted ORs*, Mat-O-Covid index categorised (95% CI
Sex						
Men	6789	415	6.11	**1**	**1**	**1**
Women	7726	602	7.79	**1.30 (1.14 to 1.48)**	**1.21 (1.03 to 1.42)**	**1.21 (1.03 to 1.42)**
Age						
<30 years	336	14	4.17	**0.47 (0.28 to 0.82)**	**0.53 (0.29 to 0.99)**	**0.52 (0.28 to 0.97)**
30–40 years	3486	338	9.7	**1.17 (1.01 to 1.36)**	**1.15 (0.97 to 1.38)**	**1.15 (0.96 to 1.37)**
40–50 years	5315	446	8.39	**1**	**1**	**1**
50–60 years	4310	177	4.11	**0.47 (0.39 to 0.56)**	**0.44 (0.36 to 0.55)**	**0.44 (0.36 to 0.55)**
>=60 years	1068	42	3.93	**0.45 (0.32 to 0.62)**	**0.43 (0.28 to 0.64)**	**0.43 (0.28 to 0.65)**
Family meeting						
No	497	24	4.83	0.66 (0.44 to 1.00)	0.66 (0.39 to 1.11)	0.66 (0.39 to 1.12)
Yes, one time	950	65	6.84	0.96 (0.74 to 1.25)	1.06 (0.77 to 1.46)	1.06 (0.77 to 1.47)
Yes, more than one time	13 023	926	7.11	1	1	1
Leisure activities						
No	3390	226	6.67	0.93 (0.80 to 1.09)	1.02 (0.84 to 1.23)	1.02 (0.84 to 1.24)
Yes, one time	836	59	7.06	0.99 (0.75 to1.30)	1.15 (0.83 to 1.60)	1.15 (0.83 to 1.60)
Yes, more than one time	10 240	730	7.13	1	1	1
Regular shopping						
No	284	19	6.69	0.95 (0.59 to 1.51)	0.94 (0.53 to 1.67)	0.94 (0.53 to 1.68)
Yes, one time	194	11	5.67	0.79 (0.43 to 1.46)	0.89 (0.45 to 1.78)	0.90 (0.45 to 1.79)
Yes, more than one time	13 984	985	7.04	1	1	1
Public places visit						
No	2254	125	5.55	**0.74 (0.61 to 0.90)**	0.84 (0.66 to 1.06)	0.84 (0.66 to 1.06)
Yes, one time	1947	135	6.93	**0.94 (0.78 to 1.14)**	0.82 (0.65 to 1.04)	0.82 (0.65 to 1.04)
Yes, more than one time	10 273	755	7.35	**1**	1	1
Use of hand sanitiser†						
No	278	20	7.19	**0.96 (0.61 to 1.53)**	**0.84 (0.45 to 1.58)**	**0.84 (0.45 to 1.58)**
Yes, after almost every outing	4696	287	6.11	**0.81 (0.70 to 0.93)**	**0.80 (0.67 to 0.95)**	**0.80 (0.67 to 0.95)**
Yes systematically after each outing (or I never go out)	9507	708	7.45	**1**	**1**	**1**
Wearring mask†						
No	380	22	5.79	0.90 (0.58 to 1.42)	0.91 (0.53 to 1.57)	0.91 (0.53 to 1.57)
Yes, after almost every outing	10 607	771	7.27	1.15 (0.99 to 1.35)	1.19 (0.98 to 1.44)	1.19 (0.99 to 1.45)
Yes, systematically after each outing (or I never go out)	3490	222	6.36	1	1	1
Difference between questionnaire and serology						
Less than 3 months	2344	212	9.04	**1**	**1**	**1**
3 months or more	12 171	805	6.61	**0.71 (0.61 to 0.83)**	**0.69 (0.57 to 0.84)**	**0.69 (0.57 to 0.83)**
Mat-O-Covid index						
Continuous variable	14 515	1017	7.01	**1.01 (1.00 to 1.02)**	**1.01 (1.00 to 1.02)**	
<13 per thousand	8950	618	6.91	**1**		**1**
≥13 per thousand	1224	116	9.48	**1.41 (1.15 to 1.74)**		**1.33 (1.07 to 1.64)**

Bold results: overall test, p<0.05.

*Adjusted for sex, age, family meeting, leisure activities, regular shopping, public places visit, use of hand sanitiser, wearing mask and time between questionnaires.

†Outside of work.

**Table 3:** Description of cases defined by ‘serology strict’ and association with bivariable and multivariable analyses based on Mat-O-Covid index (continuous and categorised)

	Total	No of cases	Proportion (%)	Crude ORs (95% CI)	Adjusted ORs*, Mat-O-Covid index continuous (95% CI)	Adjusted ORs*, Mat-O-Covid index categorised(95% CI
Sex						
Men	6804	377	5.54	**1**	**1**	**1**
Women	7758	534	6.88	**1.26 (1.10 to 1.44)**	**1.21 (1.02 to 1.43)**	**1.21 (1.02 to 1.43)**
Age						
<30 years	338	12	3.55	**0.45 (0.25 to 0.81)**	**0.47 (0.24 to 0.93)**	**0.46 (0.23 to 0.91)**
30–40 years	3499	304	8.69	**1.17 (1.00 to 1.36)**	**1.14 (0.95 to 1.37)**	**1.13 (0.94 to 1.36)**
40–50 years	5332	402	7.54	**1**	**1**	**1**
50–60 years	4323	154	3.56	**0.45 (0.37 to 0.55)**	**0.43 (0.34 to 0.54)**	**0.43 (0.34 to 0.54)**
≥60 years	1070	39	3.64	**0.46 (0.33 to 0.65)**	**0.43 (0.28 to 0.66)**	**0.44 (0.28 to 0.67)**
Family meeting						
No	499	18	3.61	**0.55 (0.34 to 0.88)**	0.48 (0.25 to 0.90)	0.48 (0.25 to 0.91)
Yes, one time	955	57	5.97	**0.93 (0.70 to 1.23)**	1.09 (0.78 to 1.52)	1.10 (0.79 to 1.53)
Yes, more than one time	13 062	835	6.39	**1**	1	1
Leisure activities						
No	3401	195	5.73	0.89 (0.75 to 1.05)	0.98 (0.80 to 1.20)	0.98 (0.80 to 1.21)
Yes, one time	841	55	6.54	1.02 (0.77 to 1.36)	1.22 (0.87 to 1.71)	1.22 (0.87 to 1.72)
Yes, more than one time	10 271	659	6.42	1	1	1
Regular shopping						
No	285	16	5.61	0.88 (0.53 to 1.47)	0.88 (0.47 to 1.64)	0.88 (0.47 to 1.64)
Yes, one time	197	8	4.06	0.63 (0.31 to 1.28)	0.75 (0.35 to 1.62)	0.75 (0.35 to 1.63)
Yes, more than one time	14 027	885	6.31	1	1	1
Public places visit						
No	2260	113	5	**0.74 (0.61 to 0.91)**	0.86 (0.67 to 1.11)	0.87 (0.68 to 1.11)
Yes, one time	1950	114	5.85	**0.88 (0.71 to 1.08)**	0.80 (0.62 to 1.03)	0.80 (0.62 to 1.03)
Yes, more than one time	10 311	682	6.61	**1**	1	1
Use o f hand sanitiser†						
No	280	18	6.43	**0.96 (0.59 to 1.56)**	**0.94 (0.50 to 1.75)**	**0.94 (0.50 to 1.76)**
Yes, after almost every outing	4710	254	5.39	**0.80 (0.69 to 0.93)**	**0.79 (0.66 to 0.95)**	**0.79 (0.66 to 0.95)**
Yes, systematically after each outing (or I never go out)	9537	637	6.68	**1**	**1**	**1**
Wearing mask†						
No	381	21	5.51	0.97 (0.61 to 1.54)	0.94 (0.54 to 1.64)	0.94 (0.54 to 1.65)
Yes, after almost every outing	10 636	690	6.49	1.15 (0.98 to 1.36)	1.20 (0.98 to 1.47)	1.20 (0.98 to 1.47)
Yes, systematically after each outing (or I never go out)	3507	199	5.67	1	1	1
Difference between questionnaire and serology						
Less than 3 months	2346	199	8.48	**1**	**1**	**1**
3 months or more	12 216	712	5.83	**0.67 (0.57 to 0.79)**	**0.65 (0.54 to 0.79)**	**0.65 (0.54 to 0.79)**
Mat-O-Covid Index						
Continuous variable	14 562	911	6.26	**1.01 (1.00 to 1.02)**	**1.01 (1.00 to 1.02)**	
<13 per thousand	8984	560	6.23	**1**		**1**
≥13 per thousand	1227	103	8.39	**1.38 (1.11 to 1.72)**		**1.30 (1.03 to 1.63)**

Bold results: overall test, p <0.05.

*Adjusted for sex, age, family meeting, leisure activities, regular shopping, public places visit, use of hand sanitiser, wearing mask and time between questionnaires.

†Outside of work.

Using the ORs obtained, we estimated proportion (and range) of COVID-19 cases attributable to work. We observed that the proportion of AFE people ranged between 20% and 40% (table 4).

**Table 4:** Attributable Fraction among people exposed (AFE) to SARS-Cov-2 at work (without and with adjustment), using the three defined outcomes

	AFE without adjustment (%)*	AFE with adjustment (%)*
‘COVID-19 reported’	43.2 (27.5 to 55.8)	36.7 (18.0 to 51.0)
‘Serology large’	29.1 (13 to 42.5)	24.8 (6.5 to 39.0)
‘Serology strict’	27.5 (9.9 to 41.9)	23.1 (2.9 to 38.7)

*The range was calculated using the upper and lower limits of the OR at 95% CI.

## Discussion

Using the COVID-19 JEM Mat-O-Covid applied to a large population, we found a significant association between work exposure to SARS-CoV-2 and COVID-19 infection, though the estimation of attributable fraction among participants exposed at work exposure was low to moderate.

This is one of the first original studies to assess the contribution of work exposure to the risk of COVID-19 infection using three validated measures to evaluate the infection. The major strength of this study is the large sample size taken from a population-based cohort with different levels of diagnosis as outcomes. Indeed, using specific and broad definitions of serology allowed us to have a more accurate picture of the breadth and spread of contamination by SARS-CoV-2. Even though misclassification due to ELISA test is possible,^9^ it is unlikely that it is a source of differential bias as it should affect all exposure groups equally. Reported results also gave similar positivity outcomes, which makes us relatively confident in the association found.

The relationship between occupations and COVID-19 was studied widely among healthcare workers^4^ whereas other jobs were considered less often. Cluster analyses in Asia identified high risk populations, such as immigrants^16^ and occupations having an increased probability of being in contact with people.^17^ These results were similar to those of other studies, with protective service occupations, administrative support occupations, education occupations, community and social services occupations, and construction and extraction occupations having the highest risk.^6^

The other relevant factors associated with COVID-19 highlight the complexity of the process of contamination. The reported extra work activities were supposed to increase the probability of COVID-19 but were found only marginally associated with COVID-19, with few significant values (family contacts). However, protective measures were not found to be inversely associated with seroconversion, but they were for reported COVID-19. The ‘almost’ category of the variable ‘use of hand sanitiser’ seemed more protective than the ‘systematically’ category, which illustrates the complexity of the interpretation of such analyses. On the other hand, older age was inversely associated with seroconversion, but not with reported COVID-19. Younger age is probably associated with lower contamination, while older age is probably associated with lower contamination and/or lower antibody production, with a possible higher proportion of immunodeficiency in older subgroups.^18–20^ A recent study using the same data, found a non-linear relationship between seroconversion and age.^10^

Some limitations to our findings exist. The sample analysed was not representative of all working age groups because CONSTANCES is not representative of the French population, and only online respondents of CONSTANCES who agreed to participate and who sent a blood test were included. However, the size of the sample and the number of different jobs (389 of the 487 existing codes) should reduce the risk of job-level selection bias. Remote work was also possible even if it was not specifically investigated here. However, one parameter on the Mat-O-Covid JEM considered remote work as a potential prevention measure. As for the exposure assessment, in general, JEMs allow us to have a sense of the group-level exposure but tend to underestimate intra-job (individual-level) variations of exposure, and Mat-O-Covid has only recently been validated.^13 21^ However, potential measurement errors caused by the JEM assessed exposure should lead to an attenuation bias and the population attributable fraction could be underestimated. Furthermore, it was necessary to check if the subjects did not change their jobs since 2020. Results with work exposure that were moderately but significantly associated with COVID-19 contamination showed that such exposure evaluations seemed to be accurate. Confounding factors that modify exposure were possible, and we could not address some of them, such as other behaviour with high risk of contamination and socioeconomic variables. Some important variables known to be associated with COVID-19, such as urban/rural habitat and household composition, were not included since they were not available for this study and since the possibility of confusion or moderation of work exposure of SARS-CoV-2 according to these variables is probably low. However, potential common variable or misclassification bias is still possible and further studies should investigate these variables to study their impact on COVID-19 work exposure. Education and social position were also not included to avoid collinearity with occupation and previous studies did not show them to be associated with COVID-19 mortality.^22^ Even if the prevalence of some comorbidities is uncommon in the working population, we could not control for residual confounding factors like immunodeficiency diseases. Finally, other locations than European countries and the evolution of new variants of SARS-CoV-2, such as the omicron variant, might give different results in the assessment of occupational exposure and other work attributable factors.

Occupational exposure represents a significant source of COVID-19 in 2020, though the associations are low to moderate. Prevention measures implemented in the workplace might explain these results and emphasise the importance of occupational health and exposure research for other COVID-19 variants and workplace risks.

## Data Availability

Data may be obtained from a third party and are not publicly available. The data of the CONSTANCES cohort are protected by our national regulatory agency ('Commission nationale de l’informatique et des libertés', no 910486). However, the CONSTANCES cohort is 'an open epidemiological laboratory' and access to study protocols and data is available on justified request.
